# Usability Factors Associated With Physicians’ Distress and Information System–Related Stress: Cross-Sectional Survey

**DOI:** 10.2196/13466

**Published:** 2019-11-05

**Authors:** Tarja Heponiemi, Sari Kujala, Suvi Vainiomäki, Tuulikki Vehko, Tinja Lääveri, Jukka Vänskä, Eeva Ketola, Sampsa Puttonen, Hannele Hyppönen

**Affiliations:** 1 National Institute for Health and Welfare Helsinki Finland; 2 Aalto University Espoo Finland; 3 University of Turku Turku Finland; 4 Helsinki University Hospital and University of Helsinki Helsinki Finland; 5 Finnish Medical Association Helsinki Finland; 6 Finnish Institute of Occupational Health Helsinki Finland

**Keywords:** health information systems, physicians, electronic health records, computers, digital

## Abstract

**Background:**

Constantly changing and difficult-to-use information systems have arisen as a significant source of stress in physicians’ work. Physicians have reported several usability problems, system failures, and a lack of integration between the systems and have experienced that systems poorly support the documentation and retrieval of patient data. This stress has kept rising in the 21st century, and it seems that it may also affect physicians’ well-being.

**Objective:**

This study aimed to examine the associations of (1) usability variables (perceived benefits, technical problems, support for feedback, and user-friendliness), (2) the number of systems in daily use, (3) experience of using information systems, and (4) participation in information systems development work with physicians’ distress and levels of stress related to information systems (SRIS) levels.

**Methods:**

A cross-sectional survey was conducted among 4018 Finnish physicians (64.82%, 2572 out of 3968 women) aged between 24 and 64 years (mean 46.8 years) in 2017. The analyses of covariance were used to examine the association of independent variables with SRIS and distress (using the General Health Questionnaire) adjusted for age, gender, employment sector, specialization status, and the electronic health record system in use.

**Results:**

High levels of technical problems and a high number of systems in daily use were associated with high levels of SRIS, whereas high levels of user-friendliness, perceived benefits, and support for feedback were associated with low levels of SRIS. Moreover, high levels of technical problems were associated with high levels of psychological distress, whereas high levels of user-friendliness were associated with low distress levels. Those who considered themselves experienced users of information systems had low levels of both SRIS and distress.

**Conclusions:**

It seems that by investing in user-friendly systems with better technical quality and good support for feedback that professionals perceive as being beneficial would improve the work-related well-being and overall well-being of physicians. Moreover, improving physicians’ skills related to information systems by giving them training could help to lessen the stress that results from poorly functioning information systems and improve physicians’ well-being.

## Introduction

### Background

The poor usability of information systems (IS)—such as problematic data entry and difficulties in use—has arisen as an important source of stress in physicians’ work [1-3]. Moreover, a recent finding shows that the physicians’ strain coming from the IS has kept rising in the 21st century [1]. Evidence implies that this strain may even affect the well-being of physicians [4,5].

Finnish physicians have given their electronic health record (EHR) systems rather critical ratings, depending on the working facility. When asked about the overall school grading for the EHR primarily in use, on a scale from 1 (*fail*) to 7 (*excellent*), the average ratings varied from 2.5 to 4.3 in 2010 and 3.2 to 4.4 in 2014 [6,7]. Recent findings from the United States showed that EHR design and use factors accounted for 12.5% of variance in measures of stress and 6.8% of variance in measures of burnout [8]. However, previous findings also showed that satisfied physicians who find their IS facilitate the continuity of care and make clinical information more accessible [9-11].

Usability problems with the current EHRs are common. Previous studies from the United States and Denmark showed over 100 usability problems, for example, related to consistency, user control, flexibility, and lack of support [12,13]. Poor IS usability and time-consuming data entry have been found as prominent sources of US physicians’ professional dissatisfaction [5]. Moreover, technical problems in IS have been related to more experiences of time pressure and lower possibilities to control one’s job [14]. Previous studies have also shown that physicians’ stress emerging from the IS is related to cognitive workload and time pressures at work [1,15].

The use of the IS is further complicated by the multiplicity of screens and options and by the need to use many different systems. There are findings suggesting that a higher number of functions increase stress and job dissatisfaction [4]. In addition, the ever-changing new functionalities and systems need constant development of physicians’ skills and time for orientation. Thus, being experienced in using different systems might help when facing challenges related to the IS.

Physicians’ participation in the development work associated with the IS might help to tackle usability problems and improve physicians’ attitudes toward the IS. However, physicians are dissatisfied with their impact possibilities and think that neither managers nor software providers are interested in end users’ opinions [16].

### Objectives

Thus, as mentioned above, the increasing use of IS in daily work is associated with many problems that stress physicians, and previous findings suggest that this might even have negative ramifications for physicians’ general well-being. However, the evidence is still limited; there are no exact findings showing which factors related to IS and EHRs are the most stressful, and whether these problems are related to the actual well-being of physicians. Therefore, this study examined the associations of (1) usability variables (perceived benefits, technical problems, support for feedback, and user-friendliness), (2) the number of systems in daily use, (3) experience of using EHRs, and (4) participation in EHR development work with physicians’ distress and stress related to IS (SRIS).

## Methods

### The Study Sample

The data were collected in April 2017 [17]. The addresses were obtained from the Finnish Medical Association’s register. A link to the study was sent via email to the target group that was all physicians younger than 65 years who lived in Finland (N=19,627). Altogether 93.37% (18,326/19,627) had provided email addresses to which the survey could be sent. The questionnaires were sent to all working-aged physicians with a cover letter calling for responses from physicians in clinical work. This was done because the Finnish Medical Association’s membership register did not allow us to select only physicians in clinical work as the target population. Thus, the sample also included physicians who were not practicing clinical patient work at the time of the data collection. Those who answered that they did not do clinical patient work (n=48) were coded as *missing*.

The representativeness of the sample was assessed by comparing the distributions of the background variables with the corresponding distribution of the target population. The respondents were slightly older than the eligible population (the percentage of those older than 54 years was 31.65% (1266/3999) in the respondents, whereas it was 26.90% (5280/19,627) in the eligible population), more often female (64.81% (2572/3968) in the respondents and 61.09% (11,992/19,627) in the eligible population), and more often specialized (67.44% (2710/4018) in the respondents and 59.90% (11,757/19,627) in the eligible population) [18]. There were no significant regional differences between the respondents and eligible population according to the place of work [18]. Due to incomplete data in some variables, the n varied between 3744 and 3780 in different analyses.

### The Context

There have been multiple reforms in Finland lately regarding IS in the health care sector. The public sector EHR adoption in Finland reached 100% in 2010, and the private sector adoption rates of EHRs are also high [19]. Finland has launched the national digital repository for electronic patient data, Kanta, in phases during the period 2012 to 2017. Kanta is targeted to health care service providers, pharmacies, and citizens. Kanta services include electronic prescriptions, My Kanta pages for citizens, a patient data repository, and an electronic prescription database. It is mandatory for all public health care providers to join Kanta and also for those private service providers that use electronic archiving.

### Measurements

The measure items used in this study can be seen in Multimedia Appendix 1.

*SRIS* was used as a dependent variable and measured with the mean of 2 items, framed in 1 question that asked how often (during the past half-year period) the respondent had been distracted by, worried about, or stressed about (1) constantly changing IS and (2) difficult, poorly performing information technology (IT) equipment or software. The answers were rated on a 5-point Likert scale ranging from 1 (*never*) to 5 (*very often*). The scale’s reliability (Cronbach alpha) was .66 in this sample. This measure has previously been used and associated with, for example, employees’ distress, cognitive workload, and higher levels of on-call duties [15,20,21]. In Finland, in addition to EHRs, a large number of separate IS are also in physicians’ use, such as laboratory and radiological data systems, clinical decision-making software, and systems related to quality, patient safety, and security [22]. The wording of this measure refers to all these systems, not only to EHRs. The reliability of this scale (.66) can be considered low but acceptable given that the scale only included 2 items [23].

*Psychological distress* was used as a dependent variable and measured with the 4 items (alpha=.84) from General Health Questionnaire-12 (GHQ-12) [24] that represent the anxiety/depression factor, as suggested by Graetz [25]. Graetz’s 3-factor structure has been suggested to be the most preferable factor model for GHQ-12 [26]. The GHQ is one of the most popular and very widely used measures of mental health and minor psychiatric disorders. A variety of scoring methods can be used when using the GHQ. The bimodal scoring method allows identification of the threshold for pathological deviations. This study used Likert-scale answer options ranging from 1 to 4 with a continuous mean variable, higher scores indicating a higher level of distress. This scoring method was used to get more variation because we were interested in general well-being and distress levels (not in pathology) in the basically healthy working-aged physician population. We have previously associated this measure with, for example, physicians’ collegial support, team climate, and patient-related stress [27,28].

The following variables were used as independent variables: *The number of systems in daily use* was assessed by asking about the number of clinical systems that the responder needed to log into on a daily basis when working with patients. The response options were 0/1/2/3/4/5 or “more”/“my work does not include clinical work” (coded as *missing*). For the analyses, this measure was coded as 0=1 to 2 systems in daily use (nobody answered that they had 0 systems in daily use) and 1=3 or more systems in daily use. *Experience of using EHRs* was assessed by asking how experienced the respondent was as an EHR user with a 5-point scale ranging from 1 (*beginner*) to 5 (*expert*). For the analyses, this variable was coded as 0=*beginner* (answer options 1-3) and 1=*expert* (answer options 4 and 5). *Participation in the development work of the IS* was assessed by asking whether respondent had participated in the development work of the IS. Answer options were as follows: *plenty*/*a little*/*no*. For the analyses, variable was coded as 0=*no* and 1=*yes* (answer options: *plenty* and *a little*).

The usability variables were used as independent variables in this study and represented the 4 strongest factors (perceived benefits, technical problems, feedback, and user-friendliness) with the highest loadings from a previous factor analysis that used 36 usability-related items among Finnish physicians [14]. These variables have previously been associated with physicians’ time pressure and control [14]. *The perceived benefits* *of the EHRs* were assessed by 6 items (alpha=.79) asking, for example, how IS help to improve the quality of care. *Technical problems* was a topic assessed by 6 items (alpha=.81), for example, “Information entered/documented occasionally disappears from the information system.” *Feedback* was assessed with 4 items (alpha=.78), such as “The system vendor implements corrections and change requests according to the suggestions of end users.” *User-friendliness* was assessed with 9 items (alpha=.86) asking, for example, whether the arrangement of fields and functions is logical on the computer screen. These usability variables were rated on a 5-point Likert scale ranging from 1 (*fully disagree*) to 5 (*fully agree*). We analyzed technical quality and user-friendliness in separate analyses to avoid multicollinearity because these variables correlated (*r*=–0.65). However, a recent validation study showed that these dimensions are separate constructs and should be studied separately as well as that all these usability variables offer a useful tool to measure the usability of the health IS [29].

The adjustment variables used were as follows: *specialization status,* which was asked as *none/specialization is ongoing/specialist. Employment sector* was categorized into 3 groups: hospitals, primary care, and other sectors. Moreover, respondents were asked their age, gender, and which EHR system they mainly use.

### Statistical Analysis

The association of independent variable levels with SRIS and distress was analyzed with analyses of covariance (in separate analyses). The analyses were conducted in 2 steps. In the first step, the analyses included adjustments variables (age, gender, employment sector, specialization status, and the EHR system in use), the number of systems in daily use, experience of using EHRs, and participation in IS-related development work. In the second step, usability variables (perceived benefits, feedback, and technical problems/user-friendliness) were added to the former model. The analyses were conducted in these 2 steps to find out whether usability variables would partly account for possible associations of the independent variables from the first step with SRIS or distress. User-friendliness and technical problems were analyzed in separate analyses to avoid multicollinearity.

## Results

### Characteristics of the Study Population

The characteristics of the study population can be seen in Table 1. The questionnaire was answered by 4018 physicians (64.82%, 2572/3968, women; response rate 21.9%) aged between 24 and 64 years (mean 46.8, SD 11.1). Almost half of the respondents worked in hospitals, and two-thirds were specialists. Over half of the respondents had 1 to 2 systems in their daily use and 71.82% (2886/4018) considered themselves as experienced in using EHRs.

**Table 1:** The characteristics of the study sample (N=4018).

Characteristic		Value
**Gender, n (%)**		
	Men	1396 (35.18)
	Women	2572 (64.82)
**Employment sector, n (%)**		
	Hospital	1943 (48.59)
	Primary health care	1070 (26.76)
	Other	986 (24.65)
**Specialist status, n (%)**		
	No	401 (10.00)
	Specialization ongoing	907 (22.57)
	Yes	2710 (67.43)
**Systems in daily use, n (%)**		
	1–2	2375 (60.43)
	≥3	1555 (39.57)
**Experience in using EHRs^a^, n (%)**		
	Beginner	1111 (27.80)
	Experienced	2886 (72.20)
**Participation in IS^b^ development, n (%)**		
	Not at all	2045 (51.34)
	Yes	1938 (48.66)
Age, mean (SD)		46.76 (11.05)
SRIS^c,d^, mean (SD)		3.32 (0.92)
Psychological distress^e^, mean (SD)		1.83 (0.66)
Perceived benefits^d^, mean (SD)		2.77 (0.79)
Technical problems^d^, mean (SD)		2.83 (0.86)
Feedback^d^, mean (SD)		2.25 (0.91)
User-friendliness^d^, mean (SD)		2.81 (0.81)

^a^EHRs: electronic health records.

^b^IS: information systems.

^c^SRIS: stress related to information systems.

^d^The scale ranged between 1 and 5.

^e^The scale ranged between 1 and 4.

### Stress Related to Information Systems

Analyses of covariance showed that all the studied variables were significantly associated with SRIS (Table 2), but the association of participation in development with SRIS attenuated to nonsignificance after adjusting for usability factors. Those who had more than 3 systems in daily use (mean SRIS 3.47, SE 0.027) had higher levels of SRIS compared with those who had only 1 or 2 systems in daily use (mean SRIS 3.23, SE 0.022; the means shown here are estimated marginal means with all adjustments). Those who had longer experience in using EHRs (mean SRIS 3.30, SE 0.022) had lower levels of SRIS compared with those who were beginners (mean SRIS 3.40, SE 0.029). High levels of technical problems were associated with high levels of SRIS, whereas high levels of user-friendliness, perceived benefits, and feedback were associated with low levels of SRIS. The study variables were able to explain much of the variance in SRIS given the rather high adjusted R squared (0.35).

**Table 2:** The results of the analyses of covariance for stress related to information systems.

Studied variables^a^	Model A		Model B	
	*F* test *(df)*	*P* value	*F* test *(df)*	*P* value
Number of systems in daily use	145.70 *(1)*	<.001	52.32 *(1)*	<.001
Experience of using EHRs^b^	12.22 *(1)*	<.001	13.73 *(1)*	<.001
Participation in IS^c^ development	15.76 *(1)*	<.001	3.54 *(1)*	.06
Perceived benefits	—^d^	—	95.13 *(1)*	<.001
Technical problems	—	—	719.50 *(1)*	<.001
Feedback	—	—	25.88 *(1)*	<.001
User-friendliness	—	—	376.86 *(1)*	<.001
R^2^	0.082 *(1)*	—	0.349 *(1)*	—

^a^All analyses were adjusted for gender, age, employment sector, specialization status, and electronic health record in use.

^b^EHRs: electronic health records.

^c^IS: information systems.

^d^Not applicable.

### Psychological Distress

The experience of using EHRs, technical problems, and user-friendliness were significantly associated with distress (Table 3). Those who were experienced users of EHRs (mean SRIS 1.82, SE 0.019) had lower levels of distress compared with those who were beginners (mean SRIS 1.92, SE 0.025). High levels of technical problems were associated with high levels of distress, whereas high levels of user-friendliness were associated with low levels of distress. Even though technical problems had a rather strong association with distress, the studied IS-related variables were not able to explain much of the variance in distress given the low adjusted R squared levels of the models.

**Table 3:** The results of the analyses of covariance for distress.

Variables^a^	Model A		Model B	
	*F* test *(df)*	*P* value	*F* test *(df)*	*P* value
Number of systems in daily use	3.61 *(1)*	.06	0.56 *(1)*	.46
Experience of using EHRs^b^	15.32 *(1)*	<.001	15.54 *(1)*	<.001
Participation in IS^c^ development	0.11 *(1)*	.75	0.00 *(1)*	.99
Perceived benefits	—^d^	—	3.74 *(1)*	.05
Technical problems	—	—	21.05 *(1)*	<.001
Feedback	—	—	0.41 *(1)*	.52
User friendliness	—	—	6.77 *(1)*	.01
R^2^	0.018 *(1)*	—	0.028 *(1)*	—

^a^All analyses were adjusted for gender, age, employment sector, specialization status, and the electronic health record in use.

^b^EHRs: electronic health records.

^c^IS: information systems.

^d^Not applicable.

## Discussion

### Principal Findings

This study found that high levels of technical problems and high number of systems in daily use were associated with high levels of IS-related stress, whereas high levels of user-friendliness, perceived benefits, and support for feedback were associated with lower levels of this stress. SRIS levels were also lower for those who considered themselves as experienced users of EHRs. Moreover, we found that IS-related variables were also associated with physicians’ well-being. More specifically, we found that high levels of technical problems were associated with high levels of psychological distress, whereas high levels of user-friendliness were associated with low distress levels. Those who considered themselves as experienced users of EHRs had lower levels of distress.

### Limitations

This study relied on self-reported measures, which may lead to problems associated with an inflation of the strengths of relationships and with common method variance. To minimize problems with self-reports, we used measures that showed good reliability and have been used in previous studies. Moreover, although we controlled for many factors—such as age, gender, employment sector, specialization status, and the EHR system in use—we cannot rule out the possibility of residual confounding. Finland is among the forerunners in the digitalization of health care [30], and tax-financed universal health care is provided for all residents; therefore, generalizing our findings to countries with other types of health care systems or IT systems should be done with caution. However, digitalization is increasing at a high pace in developed countries, and previous studies showed that IS cause stress to physicians, and all physicians have to face new challenges coming from IS [1].

The total number of respondents in the survey was rather large, about 4000. However, the response rate remained relatively low (21.92%; 4018/18,326), thus the generalizability of the findings to all physicians should be done with caution. The questionnaire was sent only electronically to physicians’ emails; thus, it was not possible to answer by paper, which may have affected the response rate. Moreover, the survey was targeted to all physicians in clinical work, but the Finnish Medical Association’s membership register did not allow us to select only physicians in clinical work as the target population. Therefore, the questionnaire was sent to all working-aged physicians with a cover letter calling for responses from physicians in clinical work. However, comparison with the target population showed good representativeness of the sample [18].

We found that IS-related variables were associated with stress levels and even well-being. However, according to our findings, it is not possible to clearly indicate whether the use of too many poorly functioning IS has extreme consequences and seriously impairs physicians’ working life. Thus, it is difficult to define the clinical meaning of our findings. Future studies are needed in this regard.

### Comparison With Previous Results

Our findings are congruent with previous findings showing that problems with IS may have negative ramifications for the well-being of physicians. For example, problems with IS have been associated with physicians’ higher likelihood of burnout [31]. Poor EHR usability, time-consuming data entry, interference with face-to-face patient care, inefficient and less fulfilling work content, an inability to exchange health information between EHR products, and the degradation of clinical documentation have all been associated with physicians’ professional dissatisfaction [5]. Moreover, technical problems in EHRs have been related to more experiences of time pressure and fewer possibilities to control one’s job [14]. Previous studies have also shown that physicians’ stress emerging from IS is related to cognitive workload, problems in teamwork, job dissatisfaction, and time pressures at work [1,15]. Moreover, IS have been associated with job dissatisfaction and intent to leave [4].

Technical problems appeared as the most important IS-related risk factor for both SRIS and psychological distress in our study. In addition, previous studies have shown the importance of the technical quality of the IS among physicians. For example, it has been shown that the technical characteristics of the IS, such as the reliability, response time, and functionality, emerged as the most important factor associated with user satisfaction [32]. Moreover, technical problems have been related to more experiences of time pressure and fewer possibilities to control one’s job [14]. Technical problems have also been found as an important barrier to the uptake of a computerized decision-support system [33]. Moreover, technical problems have also previously been found to be one of the most important challenges for patients when using mobile intervention tools [34] and Web-based intervention tools [35]. Of the technical problems, system instability in particular has been a primary concern in previous studies [6,7]. The importance of technical problems is not a surprise given that system errors, instability, missing information, low speed, and unexpected reactions may seriously challenge the workflow, waste time, hinder the doctor-patient relationship, and cause danger to patient safety.

Experience in using EHRs seemed to be an important factor in our study. Years of experience in using laboratory IS have previously been associated with usability ratings [36]. Experience is important given that systems change often, and physicians have to learn to master the new systems and are required to constantly develop their skills. In Finland, it has been found that learning to use an EHR requires a lot of training, and the time needed for this learning has increased between the years 2010 and 2014. EHRs may be challenging to use because of the multiplicity of screens, options, and navigational aids [37]. The complexity and usability problem associated with EHRs demands that physicians allocate time and effort to mastering them. However, the demands and pressures of care may not afford them this time [38]. Physicians may also see being forced to learn how to use the EHR system effectively and efficiently as a burden.

SRIS was higher among those physicians who had a higher number of systems in daily use. This corresponds well with previous findings showing that the multiple sign-ins required for multiple systems and the use of several systems simultaneously caused stress among health care professionals; in addition, the need to use multiple views was perceived as disruptive [39]. It has also been found that using several clinical systems on a daily basis led to the experience of time pressure and lessened job control [14]. We found that approximately 40% (39.56%, 1555/3930) of our respondents used 3 or more clinical systems on a daily basis. These physicians might be a group at high risk of stress. Thus, decreasing the number of systems a physician needs to log in to could have a big effect on physicians’ work-related stress levels. If it is not possible to decrease the number of systems in daily use, it might be useful to identify these physicians and offer them support or provide them with compensation for their efforts (such as extra time off).

In our study, participation in IS-related development work did not have an effect on SRIS or distress levels. Half of the participants had participated in development work, which can be considered as a big proportion. A previous study suggested that participation in development work may cause time pressure but gives an important perception of having opportunities to control one’s job [14]. It has been suggested that physicians should be included more in the development of their IS [1,15]. Moreover, it has been shown that physicians are interested in participating in IS development [16] and physician-driven improvements to EHR systems have been found to be useful [40]. An alternative approach to physicians’ participation in development work is to question why physicians should invest their time and be involved in developing the IS when their education is totally focused on another subject. On the contrary, perhaps IT professionals should invest more time and effort in understanding the needs of physicians, for example, by using robust heuristic methods and dedicated resources.

### Conclusions

We found that the usability of the IS, the number of systems in daily use, and one’s experience as a user are associated with how stressful a physician perceives the IS to be and, furthermore, to a smaller extent, associated with the physician’s well-being. According to our results, it seems that by investing in user-friendly systems with better technical quality and good support for feedback that professionals perceive as being of benefit would improve the work-related well-being and overall well-being of physicians. In particular, preventing technical problems seems to be very important.

Organizations should pay much more attention to the usability of their systems. By offering easy-to-use systems without technical problems, organizations could promote the work of physicians and release time for patient work. Good systems support workflow instead of hindering it. Improving the stability of the IS, a single sign-on, better documentation and retrieval of patient data, a peaceful documentation environment, and better access to patient data from other organizations have all been suggested as tools for promoting the IS-related well-being of health care professionals [39]. However, it seems that the needs of documenting and billing are prioritized when designing the IS instead of focusing on the needs of doctors and patients [11]. Moreover, IT professionals and hospital administrators may have a stronger voice compared with end users in decisions about the IS because they are perceived more clearly by vendors as the buyers of their systems and given higher priority [41].

Moreover, organizations would benefit from offering training opportunities to physicians and minimizing the number of their systems in daily use. However, uncertainty seems to exist about whose responsibility this training would be [39], and it would be beneficial if organizations had some kind of strategy for improving their professionals’ electronic skills. Time has been shown to be of great importance in relation to IS use [1,15]. Physicians also need some sort of support when they face IS-related problems. For instance, clerical support personnel have been found to lessen stress and fatigue after implementing new systems [42].

## Appendix

Multimedia Appendix 1 Measures used in the study.

## Abbreviations

EHR: electronic health record
GHQ: General Health Questionnaire
IS: information systems
IT: information technology
SRIS: stress related to information systems
