# Supplementary material for: Usability Factors Associated With Physicians’ Distress and Information System–Related Stress: Cross-Sectional Survey
Source: JMIR Med Inform. 2019 Nov 5;7(4):e13466. doi: 10.2196/13466 (PMC6913751; doi:10.2196/13466)
Supplement: Multimedia Appendix 1 [file medinform_v7i4e13466_app1.pdf]

APPENDIX A

Measures used in the study

Dependent variables

Stress related to information systems (SRIS):

How often has each of the issues mentioned below clearly disturbed, worried or burdened you at work during the past 6 months?

1. Constantly changing information systems
2. Difficult, poorly performing IT equipment/software

Response options:

1. Very rarely or never
2. Quite rarely
3. Every once in a while
4. Quite often
5. Very often or constantly

Psychological distress

The following questions concern your wellbeing within the last few weeks.

1. Have you often remained awake lately because of your worries?
2. Have you felt unhappy or depressed lately?
3. Have you felt constantly overburdened lately?
4. Have you felt lately that you could not cope with difficulties?

Response options:

1. Not at all
2. No more than usually
3. Slightly more than usually
4. A lot more than usually

USABILITY VARIABLES

Perceived benefits:

Use the following statements to assess how the EHR systems you use support a physician's work

1. EHR systems help to prevent medication errors.
2. EHR systems help to prevent performing duplicate examinations.
3. Use of EHR systems often takes the physician's attention away from the patient (*reverse coded*)
4. EHR systems help to safeguard the continuity of treatment.
5. EHR systems help to improve the quality of treatment.
6. EHR systems support following clinical guidelines.

Response options:

1. Completely disagree
2. Somewhat disagree
3. Don't agree or disagree
4. Somewhat agree
5. Completely agree

Technical problems:

Use the following statements to assess the usability and functionality of the EHR system from a physician's point of view

1. The system is technically stable, (no crashes, no interruptions in use)(*reverse coded*)
2. The system reacts quickly to instructions.
3. Data that have been entered sometimes disappear from the EHR system.
4. Faulty functioning of the system has caused or been close to causing a severe adverse event for a patient.

Use the following statements to assess the usability of the EHR system from a physician's point of view

5. The system often behaves unexpectedly or strangely.
6. I feel that I get enough help in problem situations associated with the use of the system (*reverse coded*)

Response options:

1. Completely disagree
2. Somewhat disagree
3. Don't agree or disagree
4. Somewhat agree
5. Completely agree

Feedback:

What kind of experiences have you had about giving feedback on the EHR systems you use and about development? Please assess the following statements based on your experiences

1. I know to whom and how I can send feedback on the system, if I so wish.
2. The system supplier is interested in feedback from users.
3. The system supplier implements suggested corrections and amendments as wished.
4. Suggestions for corrections and amendments are implemented sufficiently quickly.

Response options:

1. Completely disagree
2. Somewhat disagree
3. Don't agree or disagree
4. Somewhat agree
5. Completely agree

User friendliness:

Use the following statements to assess the usability of the EHR system from a physician's point of view.

1. Fields and functions are logically placed in the screen views (windows).
2. The system clearly announces what is happening at each stage (e.g. data being saved).
3. Terminology (such as names of functions and titles) is clear and understandable.
4. Performance of routine tasks is straightforward and can be done without extra selections.
5. Correction of certain mistakes (such as incorrect entries, ending up in the wrong view, change of selections, etc.) is easy.

Use the following statements to assess how the EHR systems you use support a physician's work.

6. Use of the EHR system does not require lengthy and thorough learning.
7. It is easy to obtain the required patient data from the EHR system.
8. Patient data can be entered easily and fluently.
9. Reminders, notes and warnings provided by the system are useful, and occur suitably frequently.

Response options:

1. Completely disagree
2. Somewhat disagree
3. Don’t agree or disagree
4. Somewhat agree
5. Completely agree

Other variables

Number of systems in daily use:

To how many clinical systems you log into on a daily basis when working with patients?

Response options: 0/1/2/3/4/5 or more/my work does not include clinical work (coded as missing).

Experience in using EHRs:

From the scale ranging from 1 (beginner) to 5 (expert), how experienced EHR user you consider yourself?

Response options:

1 = beginner

2

3

4

5 = expert

Participation in the development work on the IS:

Have you participated in development work of the IS?

Response options:

1. In plenty
2. Little
3. Not at all

Specialization status:

What is you specialization status?

Response options:

1. Not specialized
2. Specialization on-going
3. Specialist

Employment sector:

Principal occupations' employment sector

Response options:

1. Hospitals
2. Primary care
3. Private

4. University
5. Not working
